# Prognostic Factors for Thyroid Carcinoma Originating from Follicular Cells

**DOI:** 10.1155/2012/920631

**Published:** 2011-12-27

**Authors:** Yasuhiro Ito, Bryan McIver, Nikola Besic, Amit Agarwal

**Affiliations:** ^1^Department of Surgery, Kuma Hospital. 8-2-35, Shimoyamate-dori, Chuo-ku, Kobe 650-0011, Japan; ^2^Department of Endocrinology and Internal Medicine, Mayo Clinic, 200 First Street SW, Rochester, MN 55905, USA; ^3^Institute of Oncology Ljubljana, Zaloska 2, 1000 Ljubljana, Slovenia; ^4^Department of Endocrine Surgery, Sanjay Gandhi Postgraduate Institute of Medical Sciences, Raebareli Road, Lucknow 226014, India

Most thyroid carcinomas originating from follicular cells are differentiated carcinomas (DTC) that generally have an indolent character and show a favorable prognosis if treated appropriately. However, cases with certain clinicopathological characteristics are progressive and life threatening. In this special issue, scientific papers and reviews regarding the prognosis and prognostic factors of thyroid carcinoma patients were collated. 

 The first paper is a review of prognostic factors of DTC based on data from the Kuma Hospital, a hospital specializing in thyroid care. Prominent prognostic factors of DTC are indicated here in an orthodox fashion. The second one demonstrated the difference in preoperative thyrotropin serum concentrations according to the pathological diagnosis: benign nodule, papillary microcarcinoma, and clinical papillary carcinoma. It is well known that clinical node metastasis detected on preoperative imaging studies affects the prognosis of papillary carcinoma patients, as indicated in the first paper. In the third paper, the prognosis of patients showing clinical lateral node metastasis on the side contralateral but not ipsilateral to the primary lesions was investigated. Such cases generally show indolent behavior. For surgery of papillary carcinoma, it remains debatable whether central node dissection is important. The fourth paper indicates frequent metastasis to the central compartment as well as risk factors of central node metastasis and concludes that total central node dissection is preferable for papillary carcinoma.

 Most prominent prognostic factors of papillary carcinoma can be evaluated based on pre- and intraoperative findings. However, pathological examination is also important because there are some aggressive variants such as the tall cell variant of papillary carcinoma. Furthermore, small foci of anaplastic carcinoma or squamous cell carcinoma may be detected, which significantly predict a dire prognosis. However, the fifth paper demonstrated a comparatively favorable prognosis of papillary thyroid carcinoma patients with squamous cell carcinoma compartments if they can successfully undergo locally curative surgery. Papillary carcinoma shows locoregional recurrence much more frequently than distant recurrence, and the organ in which papillary carcinoma is most likely to recur is the regional lymph node. However, papillary carcinoma recurrence can lead to various other locoregional lesions. The sixth paper investigated the clinical significance of subcutaneous or intrastrap muscular recurrence and showed that it is not fatal but is a predictor of distant recurrence and that careful follow-up is required for such patients.

 The three papers from the seventh to ninth focused on up-to-date topics. The seventh one demonstrated that recombinant human thyrotropin-aided radioiodine therapy is beneficial for patients with metastatic DTC. The authors showed that this strategy yielded a therapeutic benefit in 39% of patients who otherwise could not be efficiently treated with ^131^I therapy. The eighth paper reviewed the role of ^18^FDG-PET for the diagnosis, prognosis, and management of thyroid carcinoma. The ninth paper is also a review of the *RET* rearrangement in Chernobyl-related thyroid carcinoma. This theme is quite timely because of the Tohoku disaster in Japan in March 2011.

 As indicated above, this special issue includes papers investigating the prognosis of DTC patients from various standpoints. I hope that this special issue significantly contributes to the advancement of thyroid carcinoma therapy.

*Yasuhiro Ito* *Yasuhiro Ito* *Bryan McIver* *Bryan McIver* *Nicola Besic* *Nicola Besic* *Amit Agarwal* *Amit Agarwal*

